# Supplementary material for: Chromothripsis during telomere crisis is independent of NHEJ, and consistent with a replicative origin
Source: Genome Res. 2019 May;29(5):737–49. doi: 10.1101/gr.240705.118 (PMC6499312; doi:10.1101/gr.240705.118)
Supplement: Supplemental Material [file supp_gr.240705.118_Supplemental_file_1.zip › contigs/annotated_contigs/DB45/contig.2.DB45_length_586_mean_cov_5.11945392491.docx]

**DB45_length_586_mean_cov_5.11945392491**

```
ATCACATTACACCTATAAATTGGTACAAGAATACTGTGTGATTATGATTAATTAAAATCTATAAAAGTTCTAGTTATTGTTATGTTATT
 >chr5:144142576-144142856 - E=8e-154
AATTAGTTTAATGAAGCATTAATATAAATTGAAAATATGATACTAGTAAGGTTTTAATTTTAGAAATCAATATCTCATTAGGAAGTTAC

TGCTGTATCTATAGGCATCACTAGATTAATCTCCTAGTGTAAAGGAAGCAACACCCCAAATAGCAGAAAGTACTTAGATTCATGAAGCA

TATTTATTTTTAA|CATTTCTAACAGTTATTCTAAGGACATTTAAATATTTCTGAAAATTGATAAATAATAATATTGTGATGGATGACA
               >chr5:144141306-144141615 - E=5e-167
TTTATTGAATGCCAATTAAGCTCCAAGCATTGCATTAAGTGTCTTAACTGCTTTAGCACATTTAACCTTTGCAATAACTTTATTCCAGT

AAATCCTAAATCCTATTATTAGATTGGGGGATGGGAGGTGGGGGAGGAGAGAGAGAGAGACTCAGATAGATTTAAGTAAGGTGTCCATG

TTCACACAGCTGTTTTTTTTTATTCCAAACACTGATATCGTAATTGCTATGTT
```
